# Ginsenosides and Biotic Stress Responses of Ginseng

**DOI:** 10.3390/plants12051091

**Published:** 2023-03-01

**Authors:** Paul H. Goodwin, Madison A. Best

**Affiliations:** School of Environmental Sciences, University of Guelph, Guelph, ON N1G 2W1, Canada

**Keywords:** allelopathy, disease, ginsenosides, insects, *Panax ginseng*, *Panax notoginseng*, *Panax quinquefolius*

## Abstract

Ginsenosides are saponins that possess a sugar moiety attached to a hydrophobic aglycone triterpenoid. They have been widely studied for their various medicinal benefits, such as their neuroprotective and anti-cancer activities, but their role in the biology of ginseng plants has been much less widely documented. In the wild, ginsengs are slow-growing perennials with roots that can survive for approximately 30 years; thus, they need to defend themselves against many potential biotic stresses over many decades. Biotic stresses would be a major natural selection pressure and may at least partially explain why ginseng roots expend considerable resources in order to accumulate relatively large amounts of ginsenosides. Ginsenosides may provide ginseng with antimicrobial activity against pathogens, antifeedant activity against insects and other herbivores, and allelopathic activity against other plants. In addition, the interaction of ginseng with pathogenic and non-pathogenic microorganisms and their elicitors may trigger increases in different root ginsenosides and associated gene expression, although some pathogens may be able to suppress this behavior. While not covered in this review, ginsenosides also have roles in ginseng development and abiotic stress tolerance. This review shows that there is considerable evidence supporting ginsenosides as important elements of ginseng’s defense against a variety of biotic stresses.

## 1. Introduction

Asian ginseng (*Panax ginseng*) and American ginseng (*Panax quinquefolius*) are the two most commonly cultivated species of ginseng grown for their health-promoting activities, which are primarily related to the ginsenosides that accumulate in their roots [1]. In the wild, they are slow-growing understory perennial herbs found in deciduous forests, producing a single aerial stem with 3–5 compound leaves each spring that dies back each fall, with the root overwintering [2]. This process results in a gradually enlarging root, with annual scarring of the rhizome as a result of the abscission of the aerial stem [3]. Plant age can be estimated by counting the number of scars found on the rhizome, although damage to the rhizome can create inaccuracies in age estimation due to fewer scars being visible [4]. For *P. quinquefolius*, specimens’ lifespans have been proposed to be up to an unlikely 400 years [5], but a more accurate estimate based on protected wild *P. quinquefolius* sites is between 27 and 30 years [6]. For *P. ginseng*, roots can live for several decades with the longest-lived roots found in mountain forests [7]. Thus, the roots are able to survive for long periods despite the presence of pathogens, herbivores, and competing plants, and it may be that ginsenosides play a critical role in achieving that.

## 2. Ginsenosides

Ginsenosides are a type of triterpenoid saponin, with the aglycone having a hydrophobic ring structure with carbohydrates and an aliphatic side chain attached to it [8]. Based on the ring structure, ginsenosides are classified into dammaranes, with a tetracyclic triterpene skeleton, and oleanans, with a pentacyclic triterpene skeleton [9,10]. Those with a dammarane ring structure are further categorized as ocotillols with epoxy rings attached, or protopanaxadiols (PPDs) and protopanaxatriols (PPTs), which are differentiated by an extra carboxyl group on PPDs, as well as by the location of attached sugars on the rings [10]. The code names of specific ginsenosides are mostly based on their Rf values, going from bottom to top, following separation by TLC [11]. While there are more than 100 ginsenosides that have been identified in *Panax* spp., only a few are predominant within each species [9]. The major ginsenosides in *P. quinquefolius* roots are Rb1, Rb2, Rc, Rd, Re, and Rg1, whereas the major ginsenosides in *P. ginseng* roots are Rb1, Rb2, Rc, Re, Rg1, and Rd [2,12]. In stems and leaves, the major ginsenosides are Rb3, Rd, Re, Rg1, and F11 in *P. quinquefolius*, and Rg1, Re, Rb1, Rc, Rb2, Rb3, and Rd in *P. ginseng* [13,14,15]. While the ginsenosides are similar between the two species, Rf is found in *P. ginseng* in significant amounts but in negligible amounts in *P. quinquefolius* [16]. However, F11 is found in negligible amounts in *P. ginseng* but is more significant in *P. quinquefolius*.

Ginsenosides are found in all the tissues of ginseng, including the roots, leaves, stems, and fruit [15,17]. The plants expend considerable resources to make these compounds. For example, ginsenosides in *P. quinquefolius* make up 3–7% of the root dry weight (DW) and 4–6% of the leaf DW [2,18]. Similarly, ginsenosides are 4% of the root DW and 7% of the leaf DW in *P. ginseng* species [15]. This implies that ginsenosides provide one or more important benefits to the plant, otherwise the plant would not use so many resources for producing them.

Ginsenosides are also found in the soil, but it is not known if they are actively or passively secreted from the roots. It was estimated from potted *P. quinquefolius* specimens that approximately 25 µg of F11, Rb1, Rb2, Rc, Rd, Rd, and Rg1 was secreted per day into the soil, and those were the same ginsenosides that were found in the roots [19]. Evidence for the passive secretion of ginsenosides into the surrounding soil was shown by the addition of Tween 80 or Tween 20 to a tissue culture medium, resulting in *P. ginseng* releasing approximately 76% of the total root ginsenosides into the medium [20]. This amount was significantly greater than the negligible amounts secreted without the detergents. Despite this, the amounts of ginsenosides in roots were unchanged with the detergents, indicating increased production to compensate for losses to the soil. This was attributed to the relief from the negative feedback of the ginsenoside biosynthetic pathway in the root, as well as to an increased ability to uptake nutrients into the roots. 

## 3. Biological Activities of Ginsenosides and Biotic Stresses of Ginseng 

One important role of ginsenosides appears to be as a defense against plant pathogens. Li and Utkhede [21] listed 64 fungi and 8 bacteria that were pathogenic toward *Panax* spp. The major foliar diseases of ginseng are leaf blights, which are caused by the fungi *Alternaria panax*, *Alternaria alternata*, and *Botrytis cinerea* [22]. The major root diseases are root rots caused by the fungi *Ilynocetria mors-panacis* and other *Ilynocetria* species (previously classified as *Cylindrocarpon destructans*), *Fusarium* spp., and *Rhizoctonia solani*, as well as the oomycetes *Phytophthora cactorum*, *Pythium irregulare*, and *Pythium ultimum* [22,23]. Thus, ginseng has many potential pathogens that could limit its longevity in the wild.

Ginsenosides have antimicrobial activity due to their ability to form complexes with the membrane sterols of microbial cells, which reduces membrane integrity, similar to other saponins [24]. The importance of saponins in plant disease resistance is most clearly shown by the knockout of the gene for avenacinase, a deglycosylation enzyme found in the fungus *Gaeumannomyces graminis* [25]. This enzyme is involved in the detoxification of avenacin, a saponin in oats. Avenacinase gene mutations prevented the fungus from metabolizing oat saponins and made it unable to cause root rot in oats, but it was still able to cause root rot in wheat, which does not produce avenacin. The importance of the sugar moieties as a part of saponins in terms of their toxicity was also shown for the saponins in the tree species *Sapindus mukorossi* and *Diploknema butyracea*, which had significantly reduced fungicidal activity against the fungi *Rhizoctonia bataticola*, *R. solani*, *Fusarium udum*, and *Sclerotium rolfsii* when the sugars were removed [26]. Thus, saponins can be important plant defense compounds, with their sugars appearing to be essential to their fungicidal activity. 

Although saponins are antimicrobial, their level of antimicrobial activity against different fungi can vary considerably. In fact, some fungi can have their growth stimulated by saponins. The growth of fungi in the presence of 0.10% ginsenoside from *P. quinquefolius* showed highest to lowest growth reduction in *Trichoderma harzianum*, *Trichoderma hamatum*, *A. panax*, *Trichoderma viride*, and *Fusarium solani* compared to the control [27]. *Fusarium oxysporum* showed no significant reduction, and *C. destructans* showed increased growth, perhaps due to its metabolizing the sugars on the ginsenosides.

Zhao et al. [28] found that 0.06–4.00% of total ginsenoside inhibited five fungal non-pathogens of ginseng, *Aspergillus nidulans*, *Cladosporium fulvum*, *F. oxysporum*, *Alternaria solani*, and *Alternaria porri*, but only slightly inhibited the ginseng fungal pathogen, *C. destructans*. PPT-type ginsenosides had a greater inhibitory effect than PPD types, such as with *C. destructans*, where the PPT types inhibited growth while the PPD types enhanced growth. Enhanced growth could be due to a secreted glycosidase of *C. destructans* attacking the sugars of PPD-type ginsenosides. As a result, the fungus may possibly be using the sugars as nutrients, as well as reducing toxicity, which would be similar to the β-glucosidase, avenacinase, of *G. graminis*, which reduced the toxicity of avenacin [25].

One concern in studies examining the antimicrobial effect of ginsenosides in host defenses using extracted ginsenosides in a culture medium is that such assays only test the antimicrobial activity of ginsenosides in isolation. In ginseng, many types of antimicrobial compounds are produced during the response to infection in addition to ginsenosides, such as chitinases, glucanases, protease inhibitors, benzoquinones, and phenolics [29,30,31]. Ginsenosides are likely a contributing factor to resistance that may function more to reduce the membrane integrity of a pathogen, allowing other antimicrobial compounds to be much more readily absorbed and, thus, more effective. 

Ginsenosides in soil may increase the level of root infection by attracting fungi. Nicol et al. [19] added purified ginsenosides from the soil into culture media at their estimated ecologically relevant concentrations in soil (0.06%). This stimulated the growth of the pathogenic oomycete, *P. irregulare*, but inhibited the growth of the beneficial fungus, *T. hamatum*. Thus, ginsenosides could be attracting pathogens and inhibiting non-pathogenic fungi that could act as microbial antagonists of root rot fungi. Using an in vitro diffusion assay, Ivanov et al. [32] found that Re did not affect *P. irregulare*, whereas total root ginsenoside or Rb1 inhibited hyphal growth, and F2 caused complete zones of inhibition. Soaking *P. quinquefolious* roots in purified ginsenosides before planting delayed infection by the oomycete. The authors proposed that ginsenosides temporarily stop the growth of *P. irregulare* in the rhizosphere until the organism metabolizes them, resulting in an accumulation of hyphal mass in the process. 

Ginsenosides may provide a defense against insects and other animals. The major insect pests of ginseng are cutworms (*Noctuidae* spp.), thrips (*Thrips* spp.), weevils (*Curculionidae* spp.), and wireworms (*Conoderus vespertinus*) [33]. The molecular structure of ginsenosides is very similar to that of the insect hormone, ecdysteroid, which is responsible for molting and metamorphosis; this suggests that they can be agonists to the ecdysteriod receptor, disrupting herbivorous insect life cycles [34]. Ginsenosides at 0.50%, 1.00%, and 2.00% from *P. ginseng* had an anti-feeding effect on the Northern armyworm moth (*Mythimna separate*) when feeding on *Sorghum bicolor* leaf discs soaked in ginsenosides [35]. Higher concentrations correlated with higher anti-feeding activity. Similar results were obtained against the small white butterfly (*Pieris rapae*) when *Brassica oleracea* leaves were soaked in the same range of concentrations of ginsenosides [36]. Leaf and stem extracts of *P. ginseng* contained nine different ginsenosides that had significant antifeedant activity against the diamondback moth, *Plutella xylostella*, which was proposed to be related to their ability to decrease glutathione S-transferase, acetylcholine esterase, and carboxylesterase activities in the moth [37]. In contrast, Ro, Rb1, and Rf tested negative for molluscidial activity from 2–32 ppm, unlike saponins found in other plants that have molluscidial activity [38]. In addition to herbivorous insects, large herbivores can be a major problem for ginseng leaves and stems. For example, white-tailed deer (*Odocoileus virginianus*) can completely browse the above-ground parts of 50% or more of *P. quinquefolius* plants in the wild [39]. However, there have been no studies to determine if ginsenosides have antifeedant activity against large herbivores.

The plants’ being so long-lived also opens the possibility for inter-plant competition for ginseng. One approach to such competition would be natural selection for progressively faster growth and higher reproduction rates in *Panax* spp., enabling them to quickly spread and out-compete their neighbors. However, this has not occurred. Typically, *P. quinquefolius* is found in relatively small groups of plants in the wild, rather than in large stands. Ginsenosides may contribute to this by reducing competition from other plants, including other ginseng plants, thus acting as allelochemicals. Total ginsenoside, PPD, PPT, and Re all reduced the growth of *P. ginseng* callus tissue [40]. Total, PPD, and Rb group ginsenosides had stimulatory effects at low concentrations and inhibitory effects at higher concentrations on the growth of *P. quinquefolius* seedlings [41]. For Chinese ginseng, *Panax notoginseng*, which was seeded into 0-year-old (ginsenoside-free) soil versus 1- to 3-year-old ginseng cultivation soil, seedling emergence was 93.0%, 80.0%, 60.0%, and 2.7% in 0-, 1-, 2-, and 3-year-old ginseng soils, respectively. After six months, seedling survival in ginsenoside-free soil was 78.7%, compared to 19.0% in 1-year-old soil and no survival in 2- and 3-year-old ginseng soils [42]. The concentration of total ginsenosides found in the soils increased from 0.02% to 0.04%, to 0.06% DW in 1-, 2-, and 3-year-old ginseng soils, respectively. Ginseng seedlings treated with 1 µg/g R1, Rg1, Re, Rb1, or Rd showed significantly reduced root emergence length, plant height, fresh weight (FW), and root length; however, when treated with 1 µg/g Rb3 or Rh1, only seed germination was significantly reduced with Rh1-treated seedlings. Thus, several studies have shown allelopathy due to ginsenosides, but it appears that different ginsenosides have different effects. However, there are other exudates from ginseng roots, such as phenols, that can cause allelopathy in ginseng [43]. Thus far, however, there have been no studies comparing the effectiveness and persistence of allelopathic ginsenosides with other allelopathic compounds in ginseng. 

These studies indicate that ginsenosides benefit *Panax* spp. by helping in their defense against pathogens, herbivores, and other plants. This process could be occurring within different ginseng tissues during pathogen or herbivore attacks, as well as in the soil due to secretion. It is also possible that ginsenosides are secreted onto ginseng tissue surfaces, but there are no reports of this as yet. If ginsenosides are providing benefits to the plant, then their concentrations and the relative amounts of the different ginsenosides might increase in response to various biotic stresses and related factors. There are many reports concerning the impact of different biotic stresses on the levels of ginsenosides in ginseng, and those studies are the main focus of the remainder of this review.

## 4. Effect of Pathogenic Microorganisms on Ginsenosides 

As ginsenosides may act as plant defense compounds, it is not surprising that attacks by pathogens and pests would affect ginsenoside content. The triggering of plant defense responses is related to the recognition of microbial compounds and is primarily divided into two types. Pattern-triggered immunity (PTI) is due to the recognition of pathogen-associated molecular patterns (PAMPs) or damage-associated molecular patterns (DAMPs), while effector-triggered immunity (ETI) is due to the recognition of pathogen effectors [44]. PTI is most often associated with slowing pathogen invasion, whereas ETI is most often associated with a hypersensitive response and rapid pathogen death, with extremely limited pathogen invasion, although there are exceptions. While there are a number of reports of fungi and bacteria impacting ginsenosides inside the plant, there are yet to be reports of plant viral infection or insect feeding affecting ginsenosides. 

**Figure 1:**
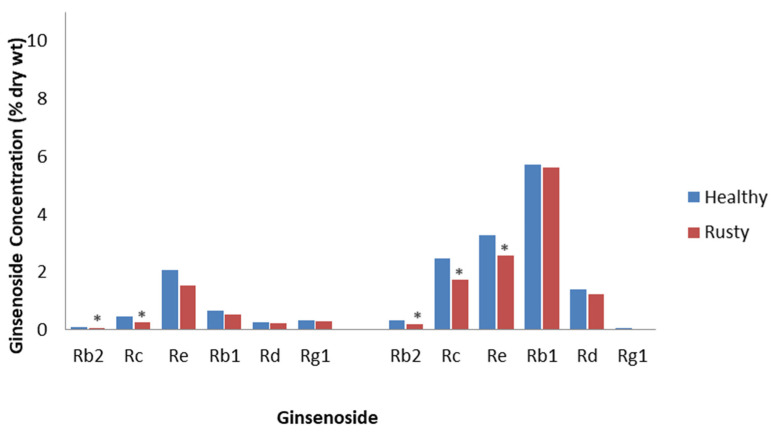
Effect of rusty root disease on specific ginsenosides. Percentage of ginsenoside by dry weight in the roots of 3- to 4-year-old *P. quinquefolius* roots collected from the field, with and without rusty root symptoms, based on the study by Rahman and Punja [47] (left), and percentage of ginsenoside by dry weight in the roots of 4-year-old *P. quinquefolius* roots collected from the field, with and without rusty root symptoms, based on the study by Campeau and Proctor [48] (right). Asterisks indicate significant differences.

Rusty root is characterized by brown lesions on the surface of roots, formed by the oxidation of Fe^2+^ to Fe^3+^ and phenols. It has been related to attacks by soil-borne bacteria, such as *Agrobacterium tumefaciens*, *Pseudomonas marginalis*, and *Rhodococcus erythropolis*, which secrete pectinases and cellulases [45]; however, another hypothesis is that it is due to incompatible interactions with the root rot fungi, *Ilyonectria robusta*, *Ilyonectria crassa*, and *Ilyonectria panacis*, which are weakly aggressive to ginseng [46]. Rahman and Punja [47] collected healthy and rusty root tissue from 3- to 4-year-old field-cultivated *P. quinquefolius* and showed that rusty roots contained 25% less total ginsenosides. Most of the ginsenosides were decreased, with the PPDs, Rc and Rb2, significantly decreased by approximately 50% (Figure 1). The reductions were proposed to be as a result of the destruction of tissue by this disease or from an immune response, as damage was only observed in the epidermis and 6 to 8 cell layers of the cortical tissues under the epidermis. This is important, as most ginsenosides accumulate in the epidermis and the underlying cortical tissue. Campeau and Proctor [48] reported that the roots of field-cultivated 4-year-old *P. quinquefolius* with rusty root symptoms had a 13% reduction in total ginsenosides compared to healthy-looking roots. All ginsenosides were significantly reduced, except Rb1 and Rg1, with the greatest reduction being a 38% decrease in Rb2 concentration compared to the control (Figure 1). They also reported that the effect of rusty root is most likely as a result of the destruction of the first few cell layers of the periderm, which is highly concentrated with ginsenosides. Considering that the host response and the invading microbes are restricted to the initial infection sites on the outer surface of the root, rusty root is one ginseng root disease that would be consistent with the definition of ETI.

**Figure 2:**
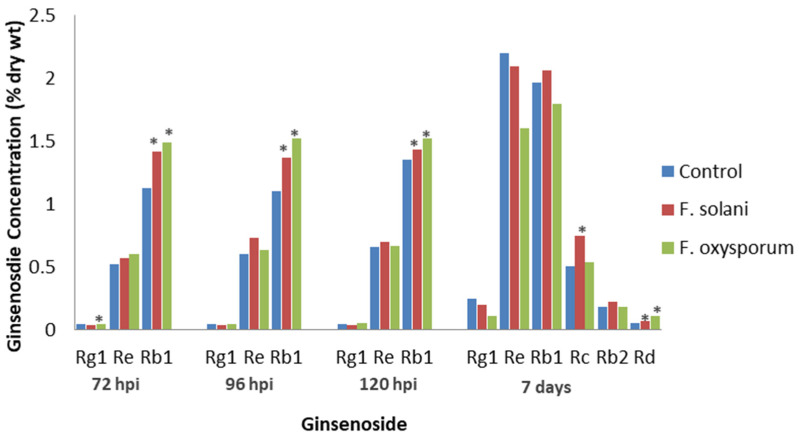
Effect of *Fusarium* diseases on specific ginsenosides. Percentage of ginsenoside by dry weight in *P. quinquefolius* roots of unknown age, either non-inoculated (control) or inoculated with *F. solani* or *F. oxysporum* at 72, 96, and 120 h post-infection, based on the study by Jiao et al. [50] (left), and the percentage of ginsenoside by dry weight in the roots of 4-year-old *P. quinquefolius* vascular tissue inoculated with distilled water (control), *F. solani*, or *F. oxysporum* at 7 days post-infection, based on the study by Jiao et al. [51] (right). Asterisks indicate significant differences.

Several *Fusarium* species cause root rot in ginseng [49]. Jiao et al. [50] inoculated cultivated *P. quinquefolius* roots of unknown age with the fungi *F. solani* or *F. oxysporum*, which had been isolated from diseased ginseng roots. The highest total ginsenoside concentration was at 120 h post-inoculation (hpi), when the water control and *F. solani-* and *F. oxysporum*-infected roots had 2.05, 2.17, and 2.24% ginsenoside DW, respectively. Re levels with both fungi and Rg1 levels with *F. solani* were not significantly different compared to the water control at 72, 96, or 120 hpi (Figure 2). In contrast, Rg1 levels were significantly lower than the control after *F. oxysporum* infection at 72 hpi, but Rb1 levels were significantly higher than the control with either *F. solani* or *F. oxysporum* infection at all time points. The results show that root infection increased ginsenoside levels, but the increase varied, depending on the fungal isolate. Among the ginsenosides tested, Rb1 was the only one to inhibit the germination of both fungi, indicating a possible role in host defense and a greater role for PPT than PPD ginsenosides in the host response. 

Jiao et al. [51] also examined the effects of *F. solani* and *F. oxysporum* infection on 4-year-old, cultivated *P. quinquefolius* roots, except that the researchers excised the vascular tissue of the roots. Both fungi were observed to infect the phloem but not the xylem. In the vascular tissue, *F. solani* infection significantly increased Rd and Rc levels, while *F. oxysporum* infection significantly increased Rd levels (Figure 2). Infection by both *Fusarium* spp. significantly decreased Rg1 levels, while the levels of Re, Rb1, and Rb2 were not significantly affected by either fungus. Compared to Jiao et al. [50], who showed that Rb1 changes were the greatest, Jiao et al. [51] noted little effect on Rb1 levels. This shows that only certain tissues are invaded during fungal infection, and ginsenoside concentrations in tissues undergoing invasion may be different from those in the whole root. 

Farh et al. [52] inoculated pots of 2-year-old *P. ginseng* with the fungi *I. robusta*, *Ilyonectria leucospermi*, and *I. mors-panacis*. Although all caused root rot in ginseng, *I. mors-panacis* was the most aggressive. Growth in vitro showed that *I. mors-panacis* was inhibited by both PPTs and PPDs, while *I. robusta* and *I. leucospermi* were most inhibited by PPTs. This indicates that PPTs may have a broader role in fungal defense. Inoculation with either *I. leucospermi* or *I. robusta* caused a significant increase in total root ginsenosides, whereas *I. mors-panacis* infection resulted in the ginsenoside concentrations being significantly lower than in the non-treated control. While the ginsenoside chromatogram profiles were shown, no concentrations of specific ginsenosides were reported. It was proposed that the reduced ginsenoside content in roots due to *I. mors-panacis* infection was not as a result of the fungus metabolizing ginsenosides in the roots for detoxification. Instead, it was due to the inhibition of the defense response (presumably PTI) by *I. mors-panacis*, thus preventing increased activities in squalene synthase, squalene epoxidase, dammarenediol-II synthase, cytochrome p450, and several glycosyltransferases that are key enzymes involved in ginsenoside synthesis, and/or via suppressing salicylic acid (SA) accumulation. This suppression of the defense responses is a characteristic of effector-triggered susceptibility, wherein the effectors suppress PTI [53]. Thus, it appears that all three *Ilyonectria* species have PAMPs to induce ginsenosides during infection, but the more aggressive *I. mors-panacis* has effectors that are able to better suppress PTI. 

In a later study, Farh et al. [54] inoculated cultivated 2-year-old *P. ginseng* with *I. mors-panacis* and *I. robusta*. Once again, they showed that infection by *I. robusta* increased root ginsenosides, while infection by *I. mors-panacis* did not. The levels of jasmonic acid (JA) were significantly increased with *I. robusta* infection, whereas SA and reactive oxygen species (ROS) were significantly increased with *I. mors-panacis* infection. Triggering SA may have suppressed the JA response; it is known that those pathways are antagonistic to each other [55]. Roots with *I. robusta* infection had an increased expression of key ginsenoside biosynthesis genes, such as squalene epoxidase, dammarenediol synthase, squalene synthase, and farnesyl pyrophosphate synthase. In contrast, roots with *I. mors-panacis* showed significantly decreased ginsenoside biosynthesis gene expression. It was proposed that the less aggressive *I. robusta* triggered an oxidative burst by NADPH oxidase, increasing JA levels and leading to increased ginsenoside production. Therefore, *I. robusta* appears to have triggered PTI and, thus, up-regulated ginsenoside production, while *I. mors-panacis* appears to have suppressed PTI and thus down-regulated ginsenoside production. 

In addition to roots, ginsenosides are found in leaves and stems [15,17], but there have not as yet been studies of their accumulation in those tissues with biotic stresses, even though there are microbial and insect pests of ginseng leaves and stems [22,33]. It is likely that ginsenoside levels change in response to the biotic stresses of above-ground tissues, similar to that in the below-ground tissues. However, there is still a need for studies examining the role of ginsenosides in the response of ginseng leaves and stems to different herbivores and pathogens, including fungi, bacteria, and viruses. 

These studies all indicate a direct relationship between ginsenosides and triggered immunity (presumably PTI) of *Panax* spp. against pathogenic microbes. During PTI, aggressive necrotrophic pathogens must detoxify the defense compounds or suppress defense responses, such as by capitalizing on JA-SA antagonism to induce one hormone response that suppresses another [56] and secreting effectors to suppress PTI [53]. The best evidence for this in ginseng is the case of *Ilyonectria* infections, where higher aggressiveness may be due to a combination of induced host SA and effectors successfully suppressing PTI, thus preventing ginsenosides from increasing, whereas lower aggressiveness may be due to there being no induction of host SA and effectors that were less successful at suppressing PTI, thus allowing ginsenosides to increase [54]. In contrast, rusty root does not show ginsenoside accumulation, but rusty root may not be due to disease susceptibility, such as root rot, but is instead the result of cell death on the root surface due to different non-pathogenic or weakly pathogenic soil microbes [44]. Perhaps this is because rusty root is more similar to the hypersensitive response on the root surface that is typically associated with ETI [57] than a disease caused by an invasive pathogen triggering PTI. This would also explain why the symptoms have a defined area and why the causal agents do not spread into the center of the root, as would be expected from a root-rot pathogen. 

## 5. Effect of Non-Pathogenic Microorganisms on Ginsenosides 

**Figure 3:**
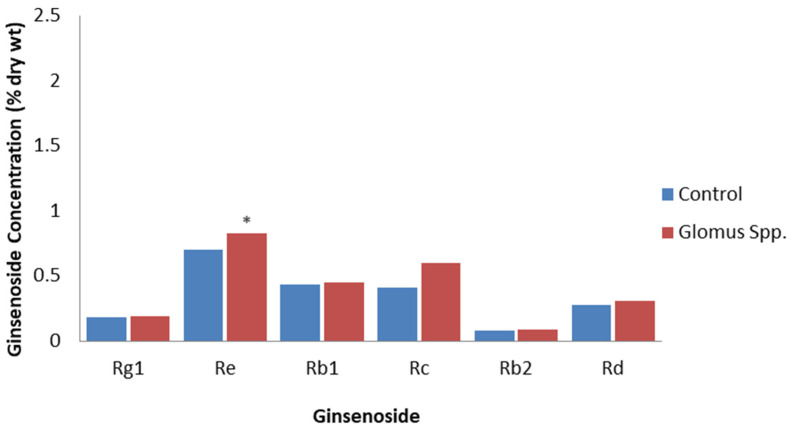
Effect of *Glomus* spp. on specific ginsenosides. Percentage of ginsenoside by dry weight in roots for the roots of 1-year-old *P. quinquefolius* roots that are non-inoculated (control) or inoculated with a mixture of spores of *G. etuticatum* and *G. intraradices* one year after inoculation, based on the study by Fournier et al. [60]. Asterisks indicate significant differences.

Non-pathogenic microbes, such as arbuscular mycorrhizal fungi and endophytes, can colonize plant tissues without causing damage, but they still have PAMPs that can induce PTI in a similar way to plant pathogens, although, generally, this is to a much-reduced extent [58,59]. A study of *P. quinquefolius* grown for one year in soil inoculated with the arbuscular mycorrhizal fungi, *Glomus etuticatum* and *Glomus intraradices*, revealed that total root ginsenosides were 18% higher than in roots grown in non-inoculated soil [60]. All the individual ginsenosides detected were higher, but only the Re levels significantly increased from 0.70% to 0.83% DW (Figure 3). The inoculation of *P. quinquefolius* seedlings with the arbuscular mycorrhizal fungi, *Rhizoglomus intraradices* or *Funneliformis mosseae*, significantly increased Rb1 levels by 26.15% and 18.43%, respectively, as well as increased Re levels by 22.38% and 18.03%, respectively [61]. On the other hand, there were no significant increases in Rg1 and Rb2 levels. The increased ginsenoside content was proposed to be due to *R. intraradices* and *F. mosseae* stimulating the expression of ginsenoside synthesis genes. Inoculation with *R. intraradices* resulted in an increase in expression of over three times for the ginsenoside synthesis genes, HMG-CoA reductase, squalene epoxidase, dammarendiol synthase, and cytochrome P450, while inoculation with *F. mosseae* increased the expression of squalene synthase, dammarendiol synthase, and cytochrome P450 genes by two to three times, compared to the non-inoculated control. However, because the arbuscular mycorrhizal infection of ginseng is beneficial for improving plant growth, plant nutrition, and soil quality [62], it is difficult to determine if the effects are due to PTI or are also due to improved plant growth and nutrition. For example, Ran et al. [54] also found that inoculation with *R. intraradices* and *F. mosseae* increased ginseng growth, photosynthetic gas exchange, stomatal conductance, and chlorophyll content. 

A correlation was found between bacterial and fungal endophyte abundance and ginsenoside content in *P. notoginseng* tissues collected from ginseng gardens. There were 89 to 221 endophytes with abundances that were significantly correlated with R1, Rg1, Re, Rb1, Rd, Rc, or Rb2 levels [63]. They noted that *Enterobacter* abundance was significantly positively correlated with R1, Rg1, Re, Rb1, and total ginsenoside levels, and *Trichoderma* and *Penicillium* abundance were significantly positively correlated with R1, Rg1, Re, Rb1, Rd, and total ginsenoside levels. As a number of endophytes could convert ginsenosides, the authors proposed that endophyte abundances were related to their ability to metabolize ginsenosides.

**Figure 4:**
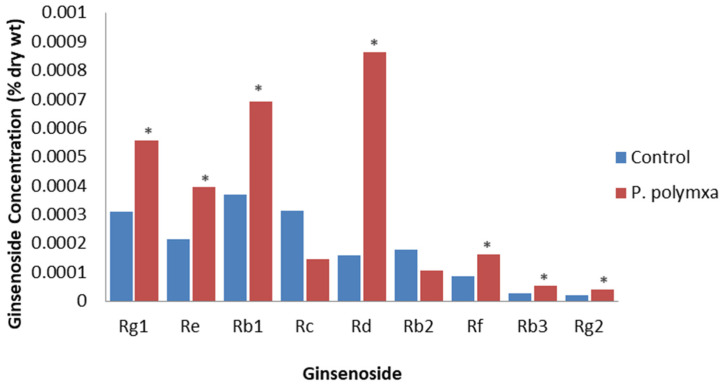
Effect of *P. polymxa* on specific ginsenosides. Percentage of ginsenoside by dry weight in the roots of 4-year-old *P. ginseng* roots inoculated with deionized water (control) or *P. polymxa* at 1 month post-inoculation, based on the study by Gao et al. [64]. Asterisks indicate significant differences.

Eight bacterial endophytes were isolated from healthy *P. ginseng* leaves [64]. The inoculation of leaves of 4-year-old plants with one of these, *Paenibacillus polymyxa*, resulted in a significant increase in total plant ginsenoside from 1.68 × 10^−3^ to 3.02 × 10^−3^ % DW, with all detectable individual ginsenosides increasing except for Rc and Rb2; the largest increase was for Rd (Figure 4). They concluded that applying this endophyte to *P. ginseng* is beneficial as it increased both yield and ginsenoside levels. It was proposed that this effect was due to the endophyte increasing JA levels.

**Figure 5:**
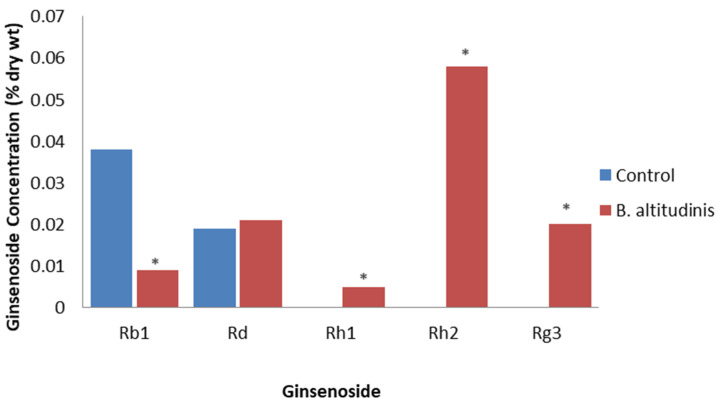
Effect of *B. altitudinis* on specific ginsenosides. Percent ginsenoside by dry weight in roots of *P. ginseng* adventitious root cultures inoculated with deionized water (control) or *B. altitudinis* at 12 days post inoculation based on the study of Song et al. [65]. Asterisks indicate significant differences.

Song et al. [65] isolated 39 endophytic bacteria from healthy field-cultivated *P. ginseng* tissue. The inoculation of adventitious roots showed that 29 isolates increased ginsenoside accumulation, but only one isolate also increased biomass, which was identified as *Bacillus altitudinis*. After determining the optimal dose at 5 × 10^8^ CFU/mL, the application of *B. altitudinis* to adventitious roots resulted in the total ginsenosides significantly increasing to 0.23% DW compared to the control at 0.07% DW. The levels of Rd were affected only slightly, but Rb1 levels were increased, as well as Rh1, Rg3, and Rh2 levels becoming now detectable, compared to being undetectable in the control (Figure 5). This study demonstrated that many different endophytic bacteria can trigger the production of ginsenosides by as much as four-fold.

Xu et al. [66] showed that inoculation with the spores of an endophytic fungus, *Chaetomium* sp., from *P. ginseng* roots could increase ginsenoside content by 3.2 times compared to a non-inoculated control in *P. ginseng* adventitious roots. Inoculation also increased the amounts of defense-related signal molecules and the expression of defense-related and ginsenoside synthesis genes, particularly farnesyl diphosphate synthase expression. This indicated that the increased ginsenosides were part of a broader defense response, possibly PTI, to the endophyte. 

The results of these studies show that non-pathogenic microbes will stimulate ginsenoside accumulation, as with the pathogenic microbes. Perhaps this is related to a shared induction of PTI increasing JA levels, resulting in the induction of gene expression for ginsenoside biosynthesis. While non-pathogenic microbes can induce ginsenosides, they can also trigger ginsenoside biosynthesis and defense-related gene expression, indicating that increased ginsenosides are part of a broader triggering of defense gene expression. Unlike pathogens, however, non-pathogenic microbes would not damage the plant. The application of these non-pathogens could be beneficial for ginseng production by increasing the value of the roots via elevating ginsenoside contents. Another benefit of applying non-pathogenic microbes is that they can cause induced systemic resistance against pathogens, which is regulated by JA and ethylene [67]. Since JA signaling is also linked with increased ginsenosides [68], it would be interesting to determine if there is a relationship between the levels of induced systemic resistance by mutualistic microbes and induced ginsenosides. 

## 6. Effects of PAMPs, DAMPs, and Effectors on Ginsenosides

As PAMPs and effectors are elements of plant pathogens, and DAMPs are partial degradation products created through the actions of a pest on the plant, it is not surprising that crude or purified compounds containing them could also trigger PTI and ETI [69]. The advantage of employing such compounds to change ginsenoside content is that they can be used without the extensive damage that can be caused by the virulence factors of the pathogen. In some cases, studies use defined PAMPs, but more often, crude extracts, termed elicitors, are used that would contain a mixture of PAMPs and other compounds. 

**Figure 6:**
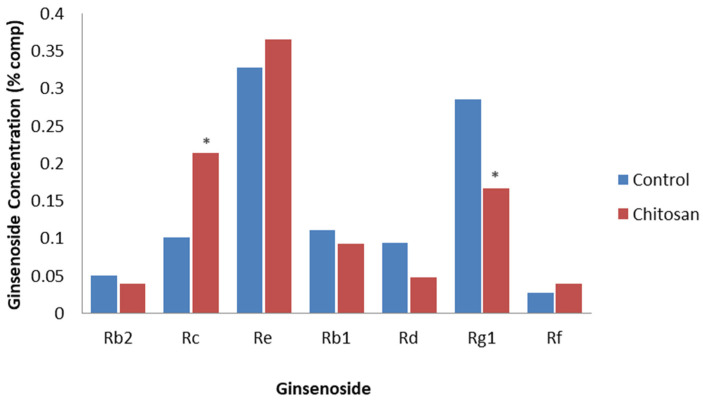
Effect of chitosan on specific ginsenosides. Percentage of ginsenoside by dry weight in the roots of 4-year-old hairy root lines of *P. ginseng*, with water (control) or chitosan treatment at 25 days post-treatment, based on the study by Palazon et al. [70]. Asterisks indicate significant differences.

Palazon et al. [70] added the PAMP, chitosan, to hairy root lines from 4-year-old *P. ginseng*. The addition of chitosan significantly reduced both biomass and total ginsenoside compared to non-treated hairy roots. After 25 days of exposure, chitosan significantly decreased the levels of PPT ginsenosides, with Rg1 declining by 17.3% of the total ginsenoside after treatment, whereas chitosan significantly increased the levels of the PPD ginsenoside, with Rc increasing to 11.6% of the total ginsenosides after treatment (Figure 6). Most of the other PPT and PPD ginsenosides only differed slightly with chitosan treatment compared to the non-treated control. This indicates a differential response of particular ginsenosides following chitosan treatment. 

**Figure 7:**
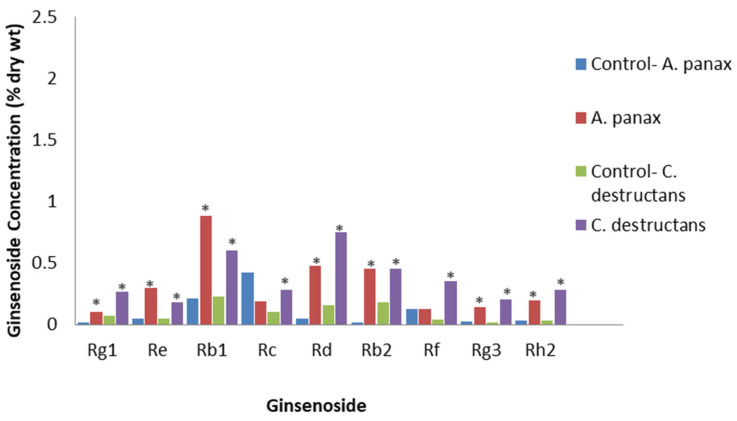
Effect of *A. panax* or *C. destructans* extracts on specific ginsenosides. Percentage of ginsenoside by dry weight in the roots of tissue culture cells of *P. quinquefolius* with no treatment (control) or a crude mycelial extract of *A. panax* or *C. destructans* at 8 days post-treatment, based on the study by Yu et al. [71]. Asterisks indicate significant differences.

A crude fungal PAMP mixture was prepared via the ultrasonic extraction of mycelium of the ginseng fungal pathogens, *A. panax* and *C. destructans* [71]. Extracts at 6 mg/L and 15 mg/L, respectively, were applied to a *P. quinquefolius* cell suspension culture derived from a callus. The levels of the stress- and defense-related signaling response compounds, nitric oxide and putrescine, were triggered, peaking at 2 and 4 days post-treatment, respectively. Ginsenoside content peaked at 8 days post-treatment, showing that nitric oxide and putrescine may be upstream regulators for ginsenoside production. The *A. panax* and *C. destructans* extracts significantly induced total ginsenoside contents to 2.10% and 2.15% DW, respectively, compared to 0.86% in the control. Although all ginsenosides were significantly increased compared to the control, except for Rc and Rf, with *A. panax* extract, the increases were greatest for the PPDs Rb1, Rd, and Rb2, and the PPT Rf (Figure 7). While it is likely that the mixture in this study contained the PAMP, chitosan, it would also contain many other PAMPs and biologically active molecules, thus making it harder to relate to studies with defined PAMPs, such as that by Palazon et al. [70].

A crude mixture of PAMPs from the mycelium of *A. panax* was also used to trigger ginsenosides in an adventitious root culture of *P. ginseng* [72]. Applying 200 mg/L of the mycelium elicitor resulted in the ginsenoside content peaking at 8 days post-treatment, with PPDs, PPTs, and total ginsenoside at 1.95%, 0.45%, and 2.40% DW, respectively. There were significant increases in the PPDs Rb1, Rb2, Rc, Rd, Rg3, and Rh2, as well as the PPTs Re, Rf, and Rg1, with the largest increases observed for Rb1, Rb2, and Rc. Both mycelium preparations and the ultrasonic extracts of mycelium were effective. These results with *P. ginseng* are very comparable to those of Yu et al. [71] with *P. quinquefolius* showing that the two *Panax* species respond similarly. 

Another study of fungal PAMPs used a soluble extract from ground mycelium or the cell-free filtrate of the non-plant pathogenic fungi, *Aspergillus niger*, *Aspergillus flavus*, and *Aspergillus oryzae* [73]. Adventitious *P. ginseng* roots were treated with these preparations, and the most effective was the mycelial extract of *A. niger*, which significantly increased total ginsenoside content to 2.99% DW. Inoculation with this extract also significantly increased the levels of JA, SA, and nitric oxide, and up-regulated ginsenoside biosynthesis genes, such as squalene synthase, squalene epoxidase, dammarenediol synthase, two cytochrome p450 genes, and three uridine diphosphate-glycosyltransferase genes. Although individual ginsenoside concentrations were not reported, it is likely that the levels of the PPD Rh2 and the PPTs Rg3 and Rh1 were increased as the expression of the uridine diphosphate-glycosyltransferase was increased, which is responsible for the biosynthesis of those ginsenosides. These results show that immunity triggered by PAMPs is correlated with increased ginsenoside accumulation in *P. ginseng* adventitious roots through defense signaling hormones and the up-regulation of gene expression.

**Figure 8:**
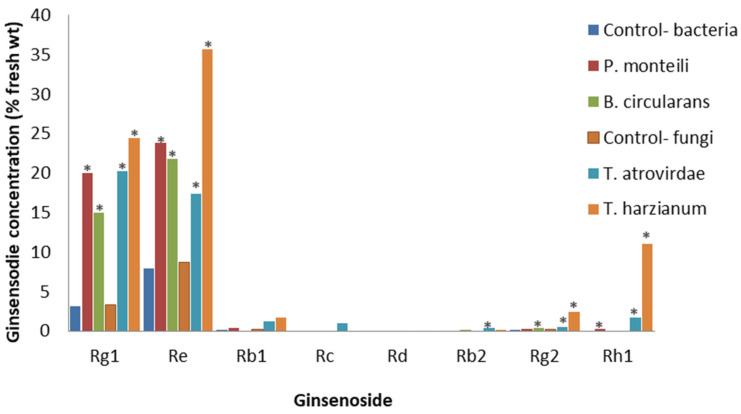
The effect of *P. monteili*, *B. circulans*, *T. atrovirdae*, or *T. harzianum* filtrates on specific ginsenosides. Percentage of ginsenoside by dry weight in the roots of root-forming callus of *P. quinquefolius* for nutrient broth (control-bacteria), potato dextrose broth (control-fungi), or 1.25 *v*/*v*% cell-free culture filtrates of the bacteria *P. monteili* and *B. circulans*, or fungi *T. atrovirdae* and *T. harzianum* at 15 days post-treatment, based on the study by Biswas et al. [74]. Asterisks indicate significant differences.

PAMPs from other non-pathogenic microbes can also affect root ginsenosides. Cell cultures of *P. quinquefolius* derived from a root callus were exposed to cell-free culture filtrates from the non-plant pathogenic soil bacteria, *Pseudomonas montelli* and *Bacillus circulans*, and the soil fungi, *T. atrovirdae* and *T. harzianum* [74]. At 15 days post-treatment, bacterial and fungal culture filtrates at either 1.25 or 2.5% significantly increased both PPDs and PPTs, but the largest changes were seen with the fungal culture filtrates. Compared to the control, the most effective was the 1.25% *T. atrovirdae* filtrate for the PPTs and the 2.5% *T. atrovirdae* filtrate for the PPDs. For the PPTs, Rg1 and Re were significantly increased with all the 1.25% bacterial and fungal filtrates, the most effective being the *T. harzianum* filtrate, while Rg2 and Rh1 were significantly increased with 1.25% *P. montelli, T. atroviridae* and *T. harzianum* filtrates, the most effective also being the *T. harzianum* filtrate (Figure 8). Increases in the PPDs also occurred, but the levels were very low and often undetectable. Based on a limited number of species, it appears that PAMPs from non-pathogenic soil fungi have stronger effects than those of non-pathogenic soil bacteria, and PPTs respond more strongly than PPDs. 

Oligogalacturonic acid is a DAMP produced through the degradation of plant cell walls during pathogen and herbivore attacks [75]. Hu et al. [76] found that the cell suspension cultures of *P. ginseng* generated H_2_O_2_ and JA at 5 min after oligogalacturonic acid exposure, reaching a maximum concentration at 16 h. The total ginsenosides significantly increased to 6.40% DW, compared to the control of 2.30%, although no specific ginsenosides were reported. Oligogalacturonic acid treatment also up-regulated the ginsenoside biosynthesis genes, squalene epoxidase and squalene synthase, similar to up-regulation by *Aspergillus* extract [73]. This shows the similarity between PTIs due to PAMPs and DAMPs.

In general, studies with PAMPs and DAMPs show very similar results to studies using living plant pathogens and non-pathogens, in terms of their effects on ginsenosides. The advantage of using PAMPs and DAMPs rather than live microorganisms is that it is a simpler system avoiding the many activities of live pathogens, such as toxins, cell wall-degrading enzymes, and effectors that could suppress PTI. However, crude PAMP preparations would include many compounds, several of which could trigger the plant’s defenses, while others could suppress the plant’s defenses. The culture conditions would also affect the types and quantities of the compounds in the preparation. The use of purified PAMP and DAMP preparations makes one more confident that this would not occur, as well as making it more likely that the results are reproducible between studies using the same materials. Future work could investigate the potential of such compounds to be developed into novel disease-control tools by inducing resistance, as well as increasing the value of roots by their having higher ginsenoside levels. 

## 7. Conclusions

**Figure 9:**
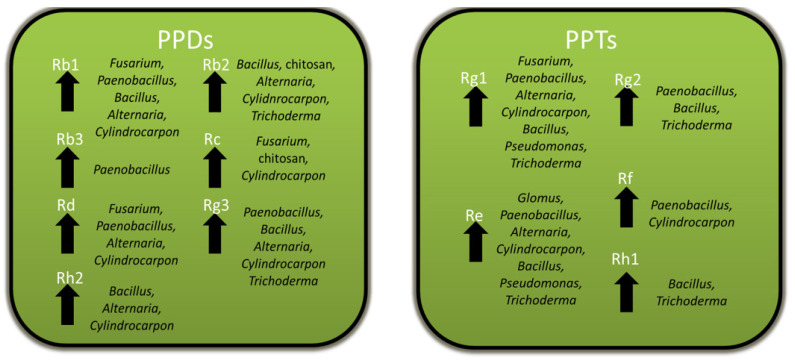
Summary of the genera of microbes or microbial products inducing significant increases in specific PPD and PPT ginsenosides. The specific ginsenoside is listed above each arrow and the genus of the microbe or microbial product is given adjacent to the arrow.

The ability of *Panax* spp. to maintain slow-growing roots that can live for many decades [5,6] may be attributed in part to the protective effects of the ginsenosides that accumulate in roots. Ginsenosides show activity against a variety of pathogens as well as other pests, such as insects and competing plants, but not all are negatively affected by them. Ginsenosides are both constitutively produced and induced by pathogenic and non-pathogenic microbes, which have been associated several times with increased ginsenoside biosynthesis and gene expression, likely as part of a broader defense response. Excluding rusty root disease, where ginsenoside decreases may be related to root cell death due to ETI, specific ginsenosides mostly increase with microbial or microbial product exposure, with Rb1, Rb2, and Rg3 being the most commonly reported increased PPDs, and Rg1 and Re being the most commonly reported increased PPTs (Figure 9). However, there is considerable variation in the specific ginsenosides affected between studies, even for basal levels in the controls. This, perhaps, should not be too surprising, as both Asian and American ginseng are difficult to breed and, thus, are genetically diverse. The most genetically uniform plants would be the *P. ginseng* cultivars that have undergone pure-line selection from local landraces [77]. Future studies should examine the variations in response between ginseng cultivars and species, to determine how that affects the response. In addition, future research should examine the relative importance of ginsenosides to other host factors during biotic stresses since ginseng would have a variety of responses [29,30,31], as in other plants. One approach could be to apply non-targeted metabolomics, which has been used to profile all detectable secondary metabolite changes during a variety of plant–microbe interactions [78]. This would permit an examination of the changes in ginsenosides relative to other changes in ginseng metabolism during infections and could also be applied to examining ginsenosides during foliar infections, a topic that has not yet been studied. 

Perhaps the greatest drawback of all the in planta studies in this review is that they are based on correlations with ginsenoside levels. This limits our conclusions about a cause-and-effect relationship between ginsenosides and the plant’s response to biotic stress. One approach to establish cause-and-effect relationships would be through the genetic modification of ginseng plants, followed by examining whether a particular response, such as a pathogen or herbivore attack, has been altered. There are reports of using the *Agrobacterium*-mediated transformation of *P. ginseng* and *P. notoginseng*, as well as using RNA interference with *P. ginseng* and *P. quinquefolius*, to examine gene function [79]. It was also proposed that CRISPR/CAS9 should be applied to the study of medicinal plants, including *Panax* spp., because of its advantages for creating gene knockouts [79]. Such genetic approaches could greatly advance our understanding of ginsenosides in the biology of ginseng plants and will eventually result in improvements in ginseng production. 

## Data Availability

No new data were created or analyzed in this study. Data sharing is not applicable to this article.
